# The speed-locking effect of particles on a graphene layer with travelling surface wave

**DOI:** 10.1186/s11671-020-03434-6

**Published:** 2020-10-28

**Authors:** Dan Wang, Lifeng Wang, Zhili Hu

**Affiliations:** Key Laboratory of Mechanics and Control of Mechanical Structures, Interdisciplinary Research Institute, College of Aerospace Engineering, Nanjing University of Aeronautics and Astronautics, Nanjing, 211100 People’s Republic of China

**Keywords:** Wave-driven mechanism, Speed-locking effect, Interaction potential, Nanoscale fast diffusion, Molecular simulation

## Abstract

Fast diffusion induced by thermal fluctuation and vibration has been detected at nanoscales. In this paper, the movement of particle on a graphene layer with travelling surface wave is studied by molecular dynamics simulation and theoretical model. It is proved that the particle will keep moving at the wave speed with certain prerequisite conditions, namely speed-locking effect. By expressing van der Waals (vdW) potential between particle and wavy surface as a function of curvatures, the mechanism is clarified based on the puddle of potential in a relative wave-frame coordinate. Two prerequisite conditions are proposed: the initial position of particle should locate in the potential puddle, and the initial kinetic energy cannot drive particle to jump out of the potential puddle. The parametric analysis indicates that the speed-locking region will be affected by wavelength, amplitude and pair potential between particle and wave. With smaller wavelength, larger amplitude and stronger vdW potential, the speed-locking region is larger. This work reveals a new kind of coherent movement for particles on layered material based on the puddle potential theory, which can be an explanation for fast diffusion phenomena at nano scales.

## Introduction

Recently, a series of surface wave/phonon-induced fast transport and diffusion phenomena are detected at micro/nanoscale. At first, the thermophoric phenomena along a carbon nanotube [1–5] or a graphene ribbon [6–10] have been extensively investigated. Thermal fluctuations are confirmed to enable continuous water flow through a carbon nanotube (CNT) by imposing an axial thermal gradient along its surface [11–13]. Nonequilibrium molecular dynamics simulations are done to explore the feasibility of utilizing a thermal gradient on a large graphene substrate to control the motion of a small graphene nanoflake [6]. In addition, thermally driven water droplet transport on graphene and hexagonal boron nitride (h-BN) surfaces is studied by molecular dynamics simulations [8, 9]. These phenomena are suggested to correlate with certain modes of phonons [14–19]. For example, Schoen et al. attributed the thermophoric motion inside a carbon nanotube to the breathing mode of the tube [1, 20]. Panizon et al. [21] pointed out flexural travelling waves/phonons on graphene can pass their momentum to the adsorbates and cause the transport. Similar to thermophoric phenomena, Angelos et al. showed temperature-induced propagating ripples on graphene can lead to fast diffusion of water nanodroplets that is 2–3 orders of magnitude faster than the self-diffusion of water molecules in liquid water [22, 23].

In addition to thermal fluctuation, studies confirm that the vibration can also transport particles and droplets in and outside a carbon nanotube (CNT) [24–27]. For example, the nanodroplets are transported along the nanotube with a velocity close to 30 nm/ns when linearly polarized transverse acoustic waves pass linear momentum to the nanodroplet [24, 28]. Guo et al. demonstrated that water molecules inside a vibrating cantilever are driven by centrifugal forces and can undergo a continuous flow from the fixed to free ends of the CNT by molecular dynamics simulations [26, 29]. A novel nanoscale unidirectional transport of water molecules through a single-walled carbon nanotube (SWCNT) is designed by using a vibration charge and a composite SWCNT with asymmetrical surface energy [30]. Zhou et al. [31] investigated current inversions in a nanosized water pump based on a single-walled carbon nanotube powered by mechanical vibration and confirmed the water current depended sensitively on the frequency of mechanical vibration. Chang and Guo [32] discovered the domino wave in carbon nanotubes which can shoot the inner molecule with a large speed up to 1 km/s. A reversible domino process is also proved in single-walled carbon nanotubes [33].

As various fast diffusion and transport phenomena induced by thermal fluctuations and vibration are detected at nanoscales, it is confirmed that the up and down movement on the surface can enhance the diffusion and transport. The connection between wave and particles’ motion is still unclear and cannot be unified. A main explanation is that the momentum of the surface can be transported to particle or droplets outside the surface [22, 24]. But the relation between amplitude, frequency and interaction between particle and surface cannot be figured out from this explanation. In addition, Angelos et al. pointed out that a clear preference for one sign of graphene curvature is necessary for fast diffusion of adsorbate on the graphene surface [22], which indicates the interaction potential induced by wavy surface morphology is closely related to the fast diffusion. Thus, exploring the interaction between wavy surface and outside particle is of essential importance to understand the mechanism of fast transport and diffusion at nanoscales.

In this paper, by studying particle outside wavy graphene surface based on vdW interaction depicted by Lennard–Jones (L–J) pair potential, a coherent relation between wavy movement and particle’s velocity is demonstrated by MD simulations. The overall speed of particle dropped onto the wavy surface is confirmed to keep the same as the travelling wave with certain prerequisite conditions, namely speed-locking effect. Then, a potential puddle theory is built based on the interaction potential between particle and wave surface expressed as a function of curvatures [34–36]. With this theory, two prerequisite conditions for speed-locking effect are proposed, and the trajectory and velocity predicted by the potential puddle theory agrees well with MD simulation results. Also, the effect of wavelength and amplitude as well as the vdW interaction parameters is analysed, which shows good agreement to the regulation detected for droplets surfing phenomena on the graphene surface [22]. The mechanism of wave-driven speed-locking effect reveals a new coherent relation between particle velocity and wavy surface.

## Methods

The MD simulation is implemented in the software package Large-scale Atomic/Molecular Massively Parallel Simulator (LAMMPS). The wavy surface is assumed to be a graphene layer, which has an atomic number density of $\rho = 3.85 \times 10^{19} \,{\text{m}}^{ - 2}$. The graphene sheet is initially flat with *z* = 0 Å and is 6344 Å long along *x* direction, resulting in a unit cell size of 6000 atoms. Along *y-axis* the periodic boundary condition is used with a period length of 12.2 Å. Here, a spherical particle is considered with the mass of $m = 0.83 \times 10^{ - 25} \,{\text{kg}}$, in order to simplify the model and focus on the geometrical effect of the wavy surface. In the beginning, the particle is placed at *z* = 7 Å and *x* = 200 Å. It has an initial speed of − 50 m/s in *z*-direction and about 2000 m/s in *x*-direction. By setting a starting time for initial speed in *z*-direction, the initial position can be controlled for the particle falling on the wavy surface.

The reactive empirical bond order (REBO) potential is adopted to model graphene atoms [37]. Meanwhile, the Lennard–Jones potential is chosen to model the interaction between particle $P$ and each carbon atom in graphene as,

1 $$u\left( R \right) = \varepsilon \left( {{\sigma \mathord{\left/ {\vphantom {\sigma R}} \right. \kern-\nulldelimiterspace} R}} \right)^{12} - \varepsilon \left( {{\sigma \mathord{\left/ {\vphantom {\sigma R}} \right. \kern-\nulldelimiterspace} R}} \right)^{6}$$

where $\varepsilon = 5.92 \times 10^{ - 21} \,{\text{J}}$ and $\sigma = 4 \times 10^{ - 10} \,{\text{m}}$. The equilibrium height between particle $P$ and the curved surface is taken as $h = 4.2 \times 10^{ - 10} \,{\text{m}}$, decided by the condition of the normal force as zero and simulation results, which is detailed in Additional file 1: 1.

The travelling wave function takes a sinusoidal form as,

2 $$y = A\sin \left( {\frac{2\pi }{\lambda }x - \omega t} \right)$$

where the amplitude is taken as $A = 1 \times 10^{ - 9} \,{\text{m}}$ and the wavelength is $\lambda = 21.75 \times 10^{ - 9} \,{\text{m}}$ unless otherwise noted. The angular frequency is taken as $\omega = {{2\pi } \mathord{\left/ {\vphantom {{2\pi } {10^{ - 12} }}} \right. \kern-\nulldelimiterspace} {10^{ - 12} }}$ corresponding to a period of 10 ps; thus, the wave speed is $v_{{{\text{wave}}}} = 2175\,{\text{m}}/{\text{s}} = \lambda \omega /2\pi$. To trigger the travelling wave, the left 10 Å of graphene (i.e. *y* $\in$ [− 10, 0] Å) is wiggled in *z*-direction with the amplitude and frequency mentioned above. Moreover, carbon atoms with *x* > 6010 Å is clamped to keep the graphene sheet stable. In particular, if a flat graphene sheet is to be simulated, unclamped graphene atoms will also be tethered to their initial positions along *z*-axis with a weak spring constant of 0.0938 eV//Å2 (besides *A* is set to 0).

An initial temperature of 5 K is assigned to unfixed carbon atoms. This temperature is set to eliminate the thermally activated ripples caused by a harmonic coupling between the bending and stretching modes of graphene and focus on the effect of the travelling wave caused by mechanical excitation [22]. The structure then evolves in micro-canonical ensemble (NVE) with a time step of 1 fs. We monitored this evolution and found temperature nearly unchanged during the whole simulation.

## Results and Discussion

**Fig. 1:**
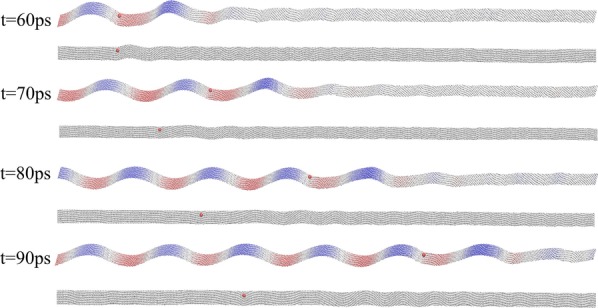
The trajectories of particle on wavy and flat graphene surfaces

The trajectory of particles on wavy graphene surface as well as flat graphene surface is illustrated in Fig. 1. The time interval is taken as the period of wavy surface. It is found that the relative position of particle does not change in reference to the wave crests or troughs, which means the particle is locked on the wavy surface with its speed equal to the wave speed. As a comparison, the particle’s overall motion on the flat surface is apparently slower than that on a wavy surface with the same initial position. The speed of particle diminishes quickly on the flat surface due to friction, whereas the friction seems not working for particles on wavy graphene surface. More MD simulation cases with different simulation temperatures and parameters of wave function are shown in Additional file 1: 1. The simulations of atom Xe and molecule C_60_ moving on wavy and flat surface are done to confirm the generalizability of this phenomenon and shown in Additional file 1: 2.

**Fig. 2:**
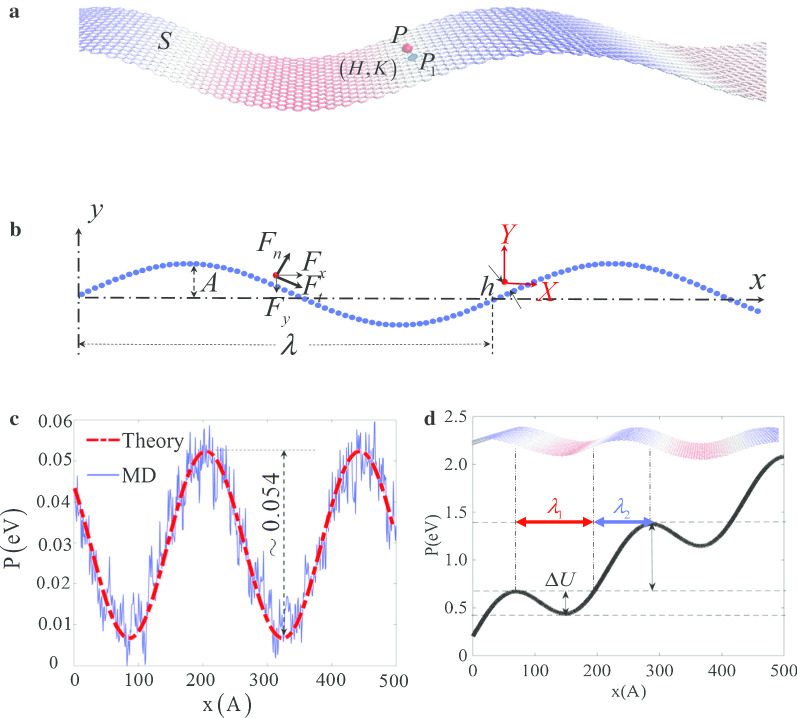
Geometry configurations and potential distribution. **a** The 3D model of wavy surface *S* and an external particle *P* with the nearest point *P*_1_ on the surface; **b** the 2D model of the wavy surface *S* and particle *P*; **c** the comparison between interaction potentials of wavy surface *S* and an external particle *P* by Eq. (1) and MD simulation; **d** the relative potential distribution in *PXY* coordinate

To understand the mechanism of speed-locking effect at nanoscales, a model is built by considering the interaction between wavy surface *S* and an external particle *P*, which is shown in Fig. 2a, b. Assuming that the wavelength and amplitude of wavy surface are $\lambda$ and *A*, respectively, the nearest height between *P* and *S* is *h*, the number density of *S* is ${\rho }_{s}$. In MD simulation, the interaction between particle *P* and wavy surface is taken as vdW interaction, which is depicted by L–J potential,

$$U_{{{\text{L}} {-} {\text{J}}}} = \varepsilon \left[ {\left( {\frac{\sigma }{r}} \right)^{12} - \left( {\frac{\sigma }{r}} \right)^{6} } \right]$$

Then, the interaction between *P* and *S* is proved to be written as a function of mean curvature and Gauss curvature based on L–J pair potential [34–36],

3 $$\begin{aligned} U_{6 - 12} & = \frac{{4\pi \rho_{s} \varepsilon \sigma^{12} }}{{5h^{10} }}\left[ {1 - hH + h^{2} H^{2} + \frac{{9h^{2} }}{16}\left( {H^{2} - K} \right)} \right] \\ & \quad - \,\frac{{2\pi \rho_{s} \varepsilon \sigma^{6} }}{{h^{4} }}\left[ {1 - hH + h^{2} H^{2} + \frac{{3h^{2} }}{4}\left( {H^{2} - K} \right)} \right] \\ \end{aligned}$$

Here, point $P_{1}$ is the nearest point on surface *S* to particle *P*, and *H* and $K$ are the mean curvature and the Gauss curvature at point $P_{1}$ (Fig. 2a) [20], respectively. Through this curvature-based potential [Eq. (3)] has been used in explaining many abnormal phenomena at micro/nano scales [38, 39], the reliability of Eq. (3) in this case is validated by compared with the surface potential in MD simulation for parameters given above and displayed in Fig. 2c.

Before analysing the influence of wavy surface on particle *P*, friction should be investigated and taken account. The friction between particles and the wave surface can be very complicated at nanoscales [39–43]. A primitive estimation of the friction is made by simulating the motion of a particle on a flat graphene layer by MD as detailed in Additional file 1: 3. For convenience, here a flat surface instead of wavy one is taken. This approximation is estimated in Additional file 1: 3 combined with further potential puddle mechanism. With parameters given above, the friction is estimated as $f = - 5.2 \times 10^{ - 13} \,{\text{N}}$.

Then, the relative potential between surface *S* and particle *P* is investigated by considering friction. Firstly, a relative wave-frame coordinate $PXY$ is built as shown in red colour in Fig. 2b, which moves at the wave speed thus keeps stationary to the travelling wave. So, the travelling wave is “frozen” in $PXY$. Since the particle keeps moving rightward in reference to the graphene, the friction acting on it will constantly be leftward along the surface. As a result, the relative wave-frame potential will be the curvature-based potential minus the work done by friction,

4 $$P = U_{n} + f * x$$

Substituting the curvature-based potential *U*_*n*_ and friction into Eq. (4), the relative wave-frame potential can be evaluated and is drawn in Fig. 2d.

Since the wave-frame coordinate *PXY* moves along with the travelling wave, the initial location of particle *P* in the potential determines the particle’s trajectory. Assuming that the initial velocity of particle *P* is $v_{0}$ and the wave speed is $v_{{{\text{wave}}}}$, two prerequisite conditions can be proposed based on Fig. 2d: the initial position of particle $P$ locates in potential puddle of the red zone $\lambda_{1}$; the initial wave-frame kinetic energy satisfies $\frac{1}{2}m\left( {v_{0} - v_{{{\text{wave}}}} } \right)^{2} \le \Delta U$. Then, the particle will not be able to hop out the puddle but instead be trapped and wobble inside the puddle. In perspective of an absolute coordinate, particle $P$ will oscillate in the potential puddle but keep moving with propagating wave by the speed locked around the wave speed, hence the speed-locking effect. Otherwise, if the initial location of particle $P$ falls inside the blue zone $\lambda_{2}$ or the relative initial kinetic energy $\frac{1}{2}m\left( {v_{0} - v_{{{\text{wave}}}} } \right)^{2} > \Delta U$, the particle $P$ will not stay inside a single puddle but hops leftward to lower puddles along the wave-frame potential surface. In the perspective of an absolute coordinate, the particle will lag behind the propagating wave until another equilibrium of forces is met. One possibility of such equilibrium is that the particle stops moving on graphene thus the friction vanishes. Interestingly, in lit [21]. Panizon et al. reveal that when there is a velocity difference, the travelling wave will be scattered by the particle and offer a propulsion force, suggesting the final speed of particle will be larger than zero.

To formulate and better illustrate our theory, the movement equation of particle P is further established by Newton's laws of motion. The driving forces exerted on particle *P* include two parts, normal force $F_{{\text{n}}}$ and tangential force $F_{{\text{t}}}$, namely (Fig. 2b),

5 $$F_{{\text{n}}} = \frac{{\partial U_{6 - 12} }}{\partial h}; \, F_{{\text{t}}} = \frac{{\partial U_{6 - 12} }}{\partial H}\nabla H + \frac{{\partial U_{6 - 12} }}{\partial K}\nabla K$$

For L–J potential, both attractive and repulsive interactions exist between atoms, the external particle $P$ will stay at a height *h* where the normal force $F_{{\text{n}}}$ is zero, the determination calculation of height *h* is put in Additional file 1: 2. Then, the equation of motion of particle $P$ in $x$ direction is,

6 $$m\ddot{x} = F_{x} - f$$

Here, $F_{x}$ is the component of tangential force $F_{{\text{t}}}$ in $x$ direction (Fig. 2b). Calculating Eq. (6) gives the particle trajectory. For the sinusoidal wave surface, Gaussian curvature is zero and the mean curvature equals to the curvature of curve in $Ozx$ surface, i.e. $K = 0$ and $H = \kappa$ [52], substituting Eq. (5) into (6), the moving trajectory of particle *P* can be solved numerically.

**Fig. 3:**
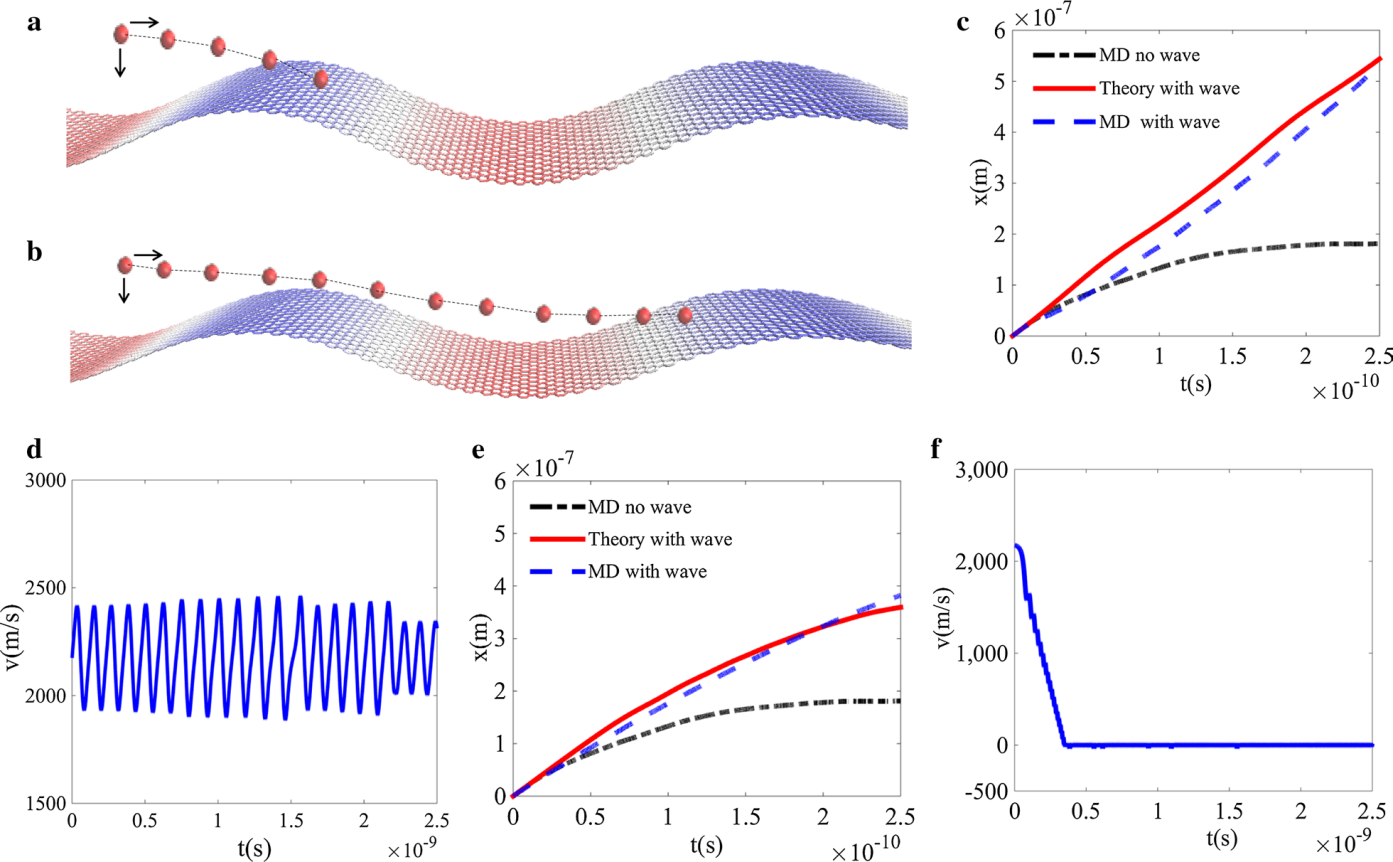
Examples of locking and unlocking. **a** A schematic view showing how the particle lands on the speed-locking region $\lambda_{1}$ of wavy graphene surface where the initial particle speed is $v_{0} = 2175\,{{\text{m}} \mathord{\left/ {\vphantom {{\text{m}} {\text{s}}}} \right. \kern-\nulldelimiterspace} {\text{s}}}$; **b** a schematic view showing how the particle lands on the speed unlocking region $\lambda_{2}$ of the wavy graphene surface; **c** the trajectories of particles by both MD simulation and theory, the trajectory of a particle on flat graphene is also plotted for comparison; **d** the time evolution of particle velocity by Eq. (6); **e** the trajectories of particle by both MD simulation and theory; **f** the time evolution of particle velocity by Eq. (6)

The locking and unlocking examples are shown in Fig. 3. For the initial location (Fig. 3a) corresponding to the locking region $\lambda_{1}$ in Fig. 2d, the trajectories from theoretical and MD simulation results are compared in Fig. 3b. It shows that the particle stops moving on the flat graphene surface in a very short time due to friction, while the particle keeps moving rightward on the wave surface. And the theoretical trajectory approximates to MD simulation results. This tendency is further confirmed in Fig. 3c for particle’s velocity shown in ten times of simulation time. Since the particle falls in the locking zone and the initial speed equals to the wave speed, it will oscillate in the potential puddle and its overall speed will equal to the wave speed, which is in accordance to our speculation. For particle with its initial location (Fig. 3d) falling in unlocking region $\lambda_{2}$ in Fig. 2d, trajectory of particle on wave surface tends to a constant (Fig. 3e) and is confirmed further by the velocity distribution. It is interesting that the travelling wave can enhance particle’s motion even when it falls on speed unlocking region compared to the motion of particle on flat graphene surface. Figure 3f illustrates that the velocity will decrease to zero for time longer than simulation time. More examples are illustrated in Additional file 1: 3.

**Fig. 4:**
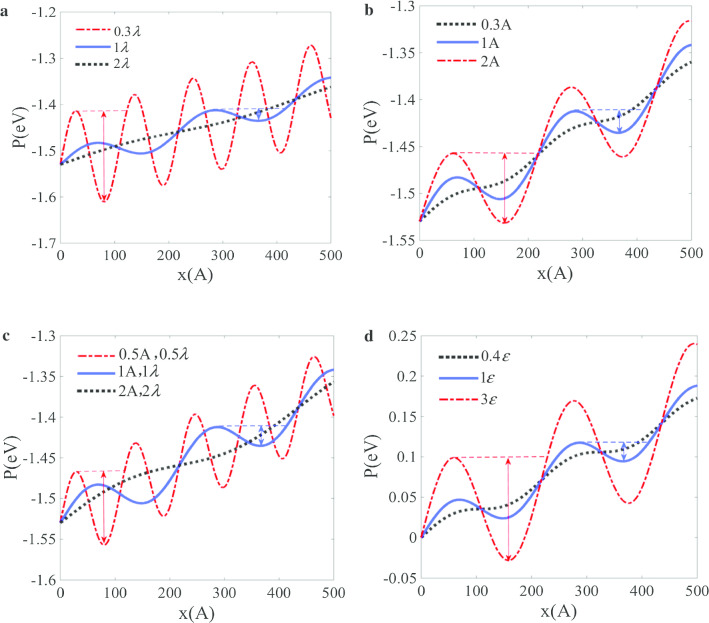
The effect of parameters on potential pubble: **a** the effect of wavelength; **b** the effect of wave amplitude; **c** the effect of ratio of wavelength and amplitude; **d** the effect of L–J potential parameter

According to the potential puddle mechanism, the speed-locking effect of particle is dominated by the potential wave surface. The effect of parameters can be discussed based on potential puddle theory. Obviously, these include wavelength $\lambda$, amplitude *A*, frequency $\omega$ and the L–J potential parameters. It is noted that the friction is assumed to remain the same regarding to different parameters in following analysis. The potential distributions for different wavelength *A*, amplitude $\lambda$ and the L–J potential parameter $\varepsilon$ are illustrated in Fig. 4, respectively. Figure 4a reveals that the potential puddle depth decreases with an increase of $\lambda$, and there will be no speed-locking range when wavelength exceeds a critical value. Also, since a lower frequency $\omega$ relates to a larger $\lambda$, the speed-locking range decreases with an increase of $\omega$. Figure 4b illustrates that the potential puddle depth increases with an increase of *A*, and the speed-locking effect vanishes when the amplitude is too small. It is noted that the ratio *A*/$\lambda$ should not be too large to prevent from damages. Usually, both $\lambda$ and *A* increase when the scale of wave or particles increases. To study the scale effect, we keep the ratio $\lambda$/*A* fixed and examine the influence of varying $\lambda$ or *A*. Figure 4c shows the potential puddle depth decrease swiftly with an increasing $\lambda$ or *A*. This indicates that the curvature-based driving force decrease quickly with an increasing scale, so the speed-locking effect for particles will vanish on surface with large-scale wave. For L–J potential parameter $\varepsilon$, it is confirmed that the speed-locking region will be broader when the pair interaction potential is strong and the speed-locking effect will disappear when the pair interaction potential is weak (Fig. 4d).

It is noted that the stiffness and L–J potential parameters are different for other 2D nanomaterials, which lead to different frequency and wave speed [44]. According to parameter analysis, the potential puddle will appear by choosing proper wavelength and amplitude for wavy surface. As the potential puddle is the precondition for particles moving with wavy surface, this speed-locking effect will also establish for many 2D nanomaterial layers under the short-range interaction.

Although the movement of one particle is discussed in this paper, it is still in the frame of thermo environment. The potential puddle is the essential condition for the coupling movement between particle and surface. For multiple particles, if they all locate in potential puddle region and satisfy the prerequisite conditions, they will be trapped and moves with the wavy surface. According to the parameters effect, the movement of particles can be controllable by adjusting the wavelength and amplitude. As the speed-locking region will be larger for surface wave with smaller wavelength, larger amplitude and higher frequency, the fast diffusion on the wavy surface will also be enhanced. The parametric analysis is also in accordance to the fast diffusion regulation detected in many other literatures. For example, Angelos et al. pointed out that the diffusion coefficient increases with the ripple amplitude of graphene surface [22]. They confirmed that the amplitude of the ripples increases revealing an increased preference of the droplets for the valleys, which can be explained by Fig. 4b. When the amplitude increases enough, the speed-locking region would probably cover the whole wavelength and enhance the diffusion. In addition, they pointed out that the potential for the valley is always smaller than the potential for crest [22] (Fig. 4), which is responding to lower potential for the crest region shown in Fig. 4. Cao et al. studied the flow of fluid inside nanochannel in the presence of travelling surface waves and confirmed that the velocity increases with the increasing of amplitude and frequency [45], which is also in accordance to the parametric analysis.

MD simulation can only reflect the property in a very short time, more potential application of this speed-locking effect can be deduced from the potential puddle mechanism. For example, by adjusting the amplitude and frequency, it is possible to realize almost locking or unlocking region, which can drive particles to move or stop. It is noted that the wavy movement of surface in vertical direction can be transformed into the movement of particle in transverse direction, which is similar to a kind of ratchet movement and may be used in nano-electro-mechanical system. In addition, as the interaction between particle and surface will affect the movement, the trajectory enhanced by wavy surface will be different for particles with different pair potentials, which can lead to phrase separation.

## Conclusions

In conclusion, we demonstrate a distinctive relation between particle and graphene layer with travelling surface wave, i.e. speed-locking phenomenon. By MD simulation, it confirmed that the speed of particle can be kept around wave speed with certain conditions. A theoretical model is built to elucidate the mechanism, where the puddle of potential surface dominates the locking effect. The locking conditions are proposed based on this model, i.e. the initial position of particle locates in the potential puddle and initial kinetic energy cannot drive particle to hop out of potential puddle. The particle trajectory predicted by theoretical predictions agrees well with MD simulations results. The effect of wavelength and amplitude as well as L–J potential parameter is discussed. The work also provides a new perspective to understand the fast diffusion and transport on wavy surface and potential applications for phrase separations.

## Supplementary information

**Additional file 1.** More simulation results with different parameters and particles.

## Data Availability

All data generated or analysed during this study are included in this published article [and its Additional files].

## Abbreviations

MD: Molecular dynamics
vdW: Van der Waals
CNT: Carbon nanotube
h-BN: Hexagonal boron nitride
SWCNT: Single-walled carbon nanotube
L–J: Lennard–Jones
LAMMPS: Large-scale Atomic/Molecular Massively Parallel Simulator
REBO: Reactive empirical bond order
NVE: Micro-canonical ensemble
