# Supplementary material for: The speed-locking effect of particles on a graphene layer with travelling surface wave
Source: Nanoscale Res Lett. 2020 Oct 28;15:203. doi: 10.1186/s11671-020-03434-6 (PMC7593379; doi:10.1186/s11671-020-03434-6)
Supplement: Supplementary file 1 — Additional file 1. More simulation results with different parameters and particles. [file 11671_2020_3434_MOESM1_ESM.docx]

**Additional file**

**1 More simulation results with different parameters**

In MD simulation, the temperature and wave amplitude have been changed to confirm the generality of speed-locking effect. It is noted that the speed-locking effect will disappear when temperature is high as the graphene surface will have obvious thermal vibration in MD simulation. Thus, this effect is confirmed when temperature is set to 25K, 50K and 100K as shown in **Fig.A1**. The speed-locking effect is also detected when wave amplitude is 0.9nm, but disappears when wave amplitude decreases to 0.5nm (**Fig.A1**).

**
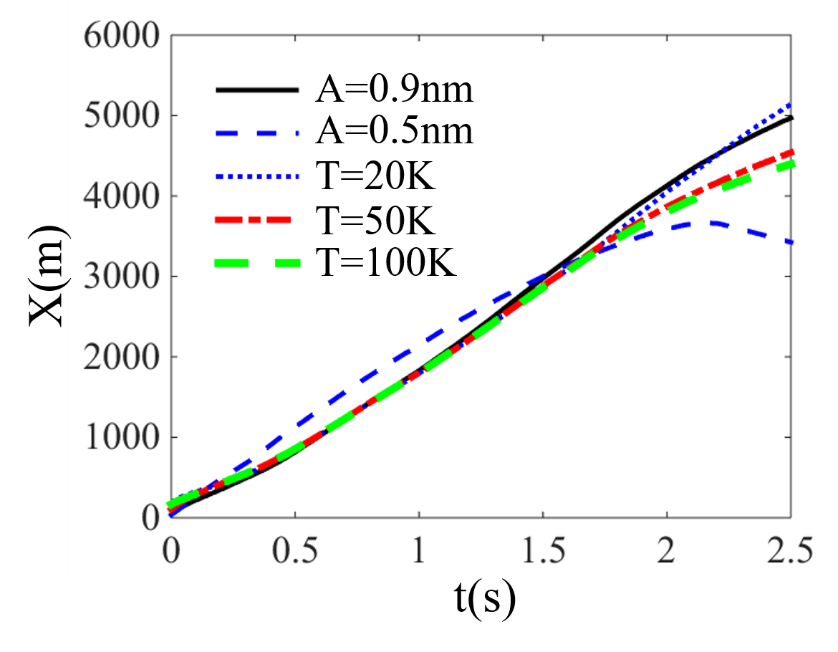
**

**Fig.A1** The trajectory of particle on the wavy graphene surface with different wave amplitudes and temperatures

**2 More simulation results with different particles**

As the spherical particle is discussed in the paper, we choose an atom Xe and molecule C_60_ to confirm the generality. As the interaction potential between particle and graphene surface will change, some parameters will also change here. For C_60_, the REBO potential is used and the initial speed is set to 1600m/s. For Xe atom, the REBO potential is used for graphene and the L-J potential between Xe and graphene is defined by MD command hybrid with $\varepsilon=3.34\times10^{\text{-}21}J$ and $\sigma=5.38\times10^{\text{-10}}m$. The initial speed is set to 1400m/s. Other parameters are the same to simulation parameters presented in the paper. The moving trajectories are shown in **Fig.A2** by comparing the trajectory on wavy surface with one on flat surface.


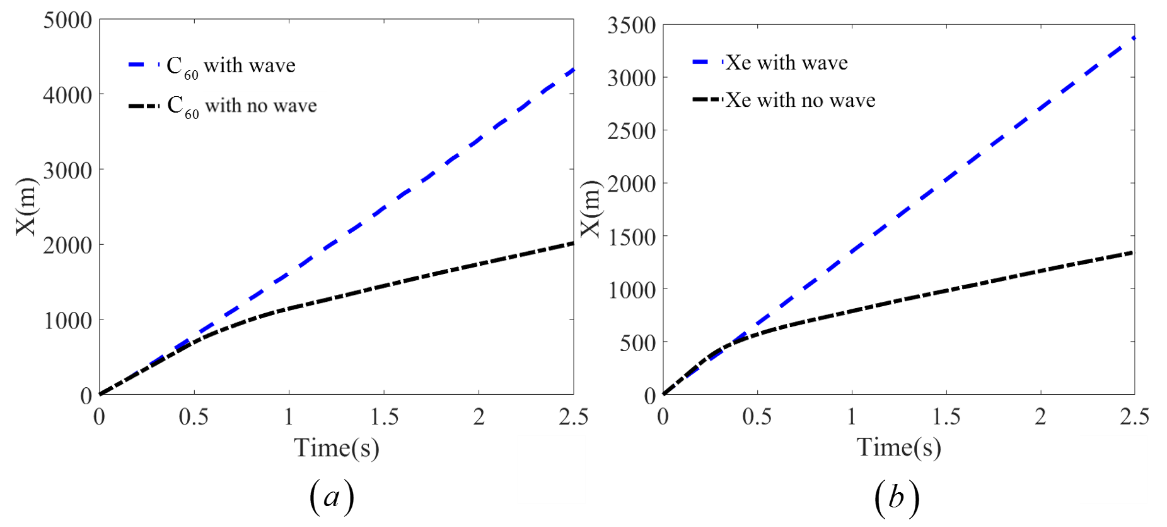


Fig.A2 The trajectory of specific particles on graphene surface: (a) The trajectory of C_60_ on wavy and flat graphene surface; (b) The trajectory of Xe on wavy and flat graphene surface.

**3 The equilibrium height of particle above surface with L-J potential**

The normal force between particle $P$ and the curved surface is,

$F_{n}=\frac{8\pi\rho_{s}C}{\left(n-2\right)h^{n-2}}\left[\frac{n-2}{h}-\frac{1}{\left(n-3\right)}H+\frac{h}{\left(n-4\right)}H^{2}+\frac{n-3}{\left(n-4\right)^{2}}\frac{h}{2}\left(H^{2}-K\right)\right]\text{ }n\geq3$ (A1)

For L-J potential $u\left(R\right)=\varepsilon\left(\sigma/R\right)^{12}-\varepsilon\left(\sigma/R\right)^{6}$, the equilibrium height $h$ of particle must satisfies the condition that the normal force between the particle and the substrate is zero, i.e. $F_{6}-F_{12}=0$.

Since the curvature of wavy surface keeps changing, the real-time height $h$ is not a constant, it is noted that the curvature of surface surely affect the real height, but the effect is small in our case,

$\frac{8\pi\rho_{s}\varepsilon\sigma^{12}}{h^{9}}-\frac{8\pi\rho_{s}\varepsilon\sigma^{6}}{h^{3}}=\left(\frac{4\pi\rho_{s}\varepsilon\sigma^{12}}{45h^{9}}-\frac{2\pi\rho_{s}\varepsilon\sigma^{6}}{3h^{3}}\right)Hh$ (A2)

In Eq.(A2), $Hh\sim\left(A/\lambda\right)*\left(h/\lambda\right)$ is a small quantity as wave length $\lambda$ is much larger than wave amplitude *A* and height *h*. Thus the left term in Eq.(A2) is omitted, a simplification is made that the equilibrium height between the particle and the wavy surface approximates to one between a particle and a flat surface, which is

$\frac{8\pi\rho_{s}\varepsilon\sigma^{12}}{h^{9}}-\frac{8\pi\rho_{s}\varepsilon\sigma^{6}}{h^{3}}=0$ (A3)

The solution of Eq.(A3) is $h=\sigma$. The value of height $h$ in the work is taken by considering the data in MD simulation and the curved based potential.

**4 The friction of particles on wavy graphene**

The friction at nanoscale is very complex and although many models have been proposed to depict it, they may not be suitable for the current work due to the waviness of substrate. In order to estimate the friction, MD simulations are employed.

The graphene substrate is initially flat and is 6344Å along *y* direction, while along *x-axis* the periodic boundary condition is used with a period length of 4.9 Å. As a result within one period the graphene has 6000 atoms. The left 10Å of graphene is wiggled in *z-axis* with an amplitude of 10 Å and a period of 10 fs. This triggers the travelling wave on graphene. Moreover, carbon atoms at *x*>6010 Å is clamped to keep the graphene stable.

In the begging the particle is placed at 7 Å above the mass center of graphene and 210 Å right to the wiggled region of graphene. It has an initial speed of -50m/s along *z*-axis and about 2000 m/s along *x*-axis.

The Reactive Empirical Bond Order (REBO) potential is adopted for graphene [45]. While for the interaction between the graphene and the particle, the LJ 6-12 potential is used as in Eq.(**A4**). An initial temperature of 5K is assigned to unfixed atoms. It then evolves according to the NVE ensemble. We monitored this evolution and noticed temperature is basically unchanged during the whole simulation. Time step is taken as 1 fs. The particle trajectory is recorded once per 10 fs. The simulation is implemented in the software package Large−scale Atomic/Molecular Massively Parallel Simulator (LAMMPS).

With the parameter set given in this work, we simulate the motion of a particle on a flat graphene with an initial speed of $2175m/s$. The trajectory is plotted in **Fig.A3**, a quadratic polynomial function is used and shown well fitting with the trajectory,

$x=at^{2}+bt+c$ (A4)


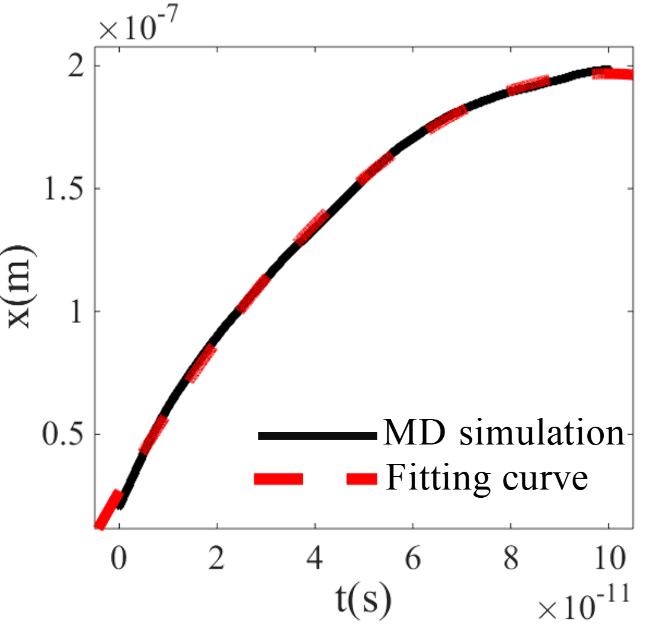


**Fig.A3** The trajectory of a particle on graphene with an initial velocity of $2175m/s$ and the fitting curve.

Then the friction can be derived as

(A5)

More MD simulations are done which confirm that the effect of initial velocities of particle on friction is negligible.

As mentioned above, the friction between particles and the wavy surface is very complex, it is simplified as the friction between particles and a flat surface in the paper. Actually, the friction between wavy surface and an external particle may not be equal to the one on flat surface and even not a constant. In order to neglect the error brought by the approximated friction, a series of MD simulations is done to figure out locking and unlocking region on the wavy surface by changing the initial locations of particle, which is shown in **Fig.A4**.


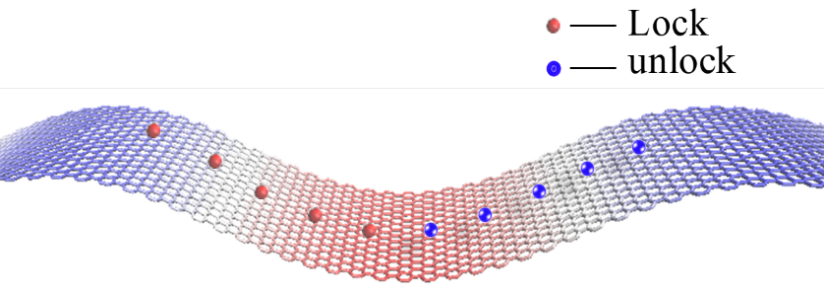


**Fig.A4** The lock and unlock zone in MD simulation for the parameters in the paper

**Fig.A4** shows that the locking region by MD simulation is a little narrow compared to the locking zone predicted by the theory with constant friction, which means the actual friction is larger than the approximated one. As the difference is not very significant, we still use a constant to analyze in the paper.

**5 More examples for speed-locking and unlocking phenomena**

In the paper, we have given the conditions for speed-locking and unlocking phenomena, more examples are shown in **Figs.A5** to **A8** to verify these conditions.


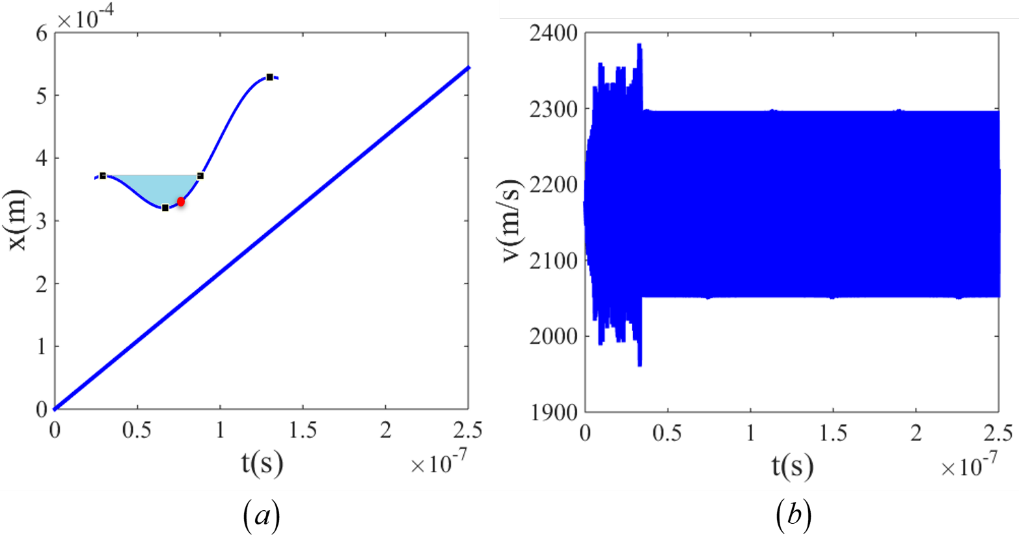


**Fig.A5** An example of speed-locking when *v*_0_=*v_wave_* (a) The initial position of particle $P$ on the potential surface and the trajectory; (b) The locking velocity of particle $P$ with initial velocity $v_{0}=2175m/s$


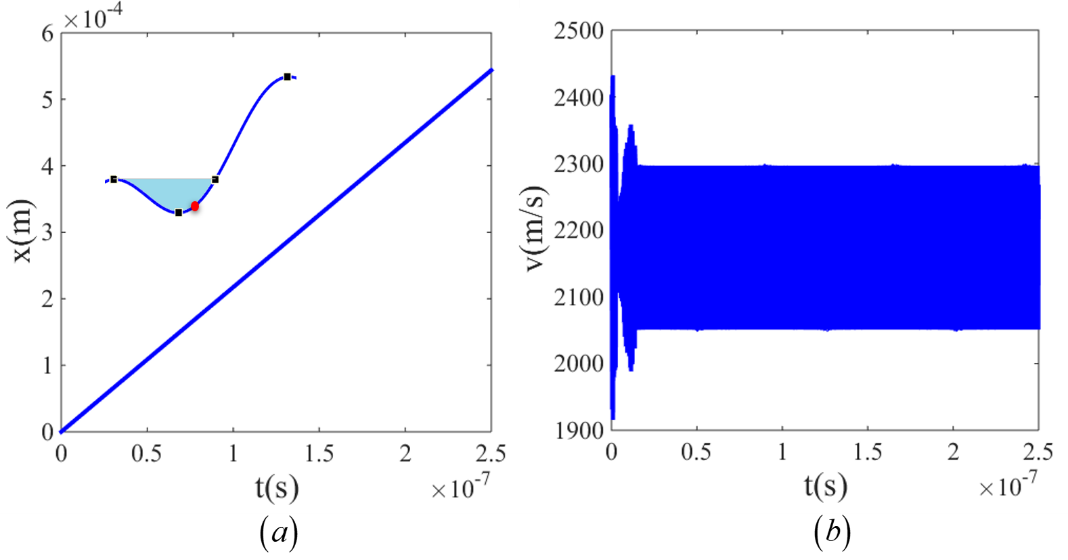


**Fig.A6** An example of speed-locking when *v*_0_≠*v_wave_*. (a) the initial position of particle $P$ on potential surface and the trajectory; (b) the locking velocity of particle $P$ with initial velocity $v_{0}=2400m/s$


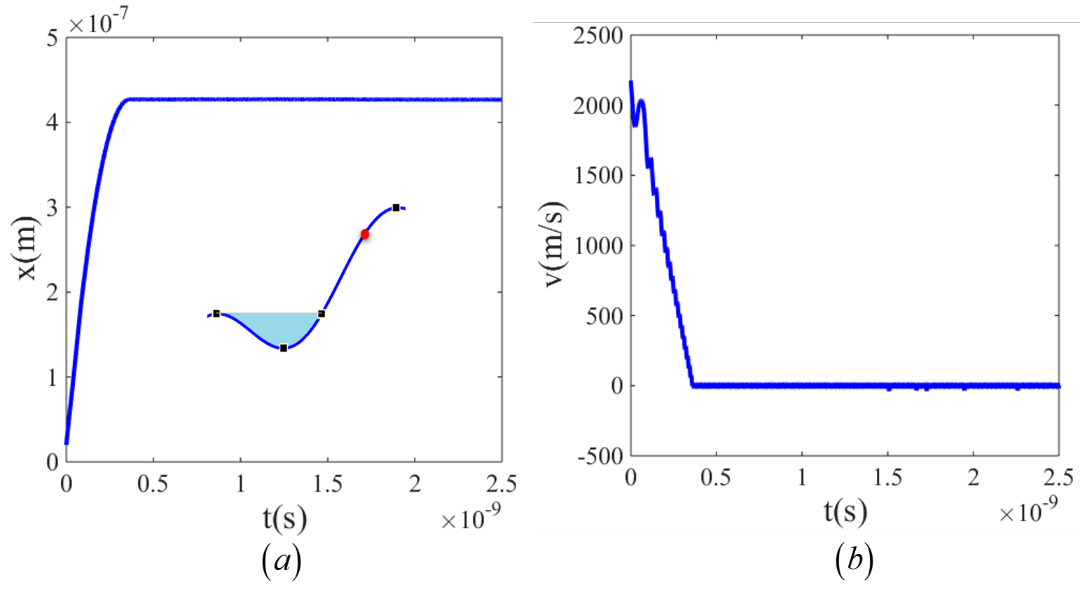


**Fig.A7** An example of the unlocking of particles when *v*_0_=*v_wave_*. (a) The initial position of particle $P$ on the relative potential surface and the trajectory; (b) The unlocking velocity of particle $P$ with initial velocity $v_{0}=2175m/s$


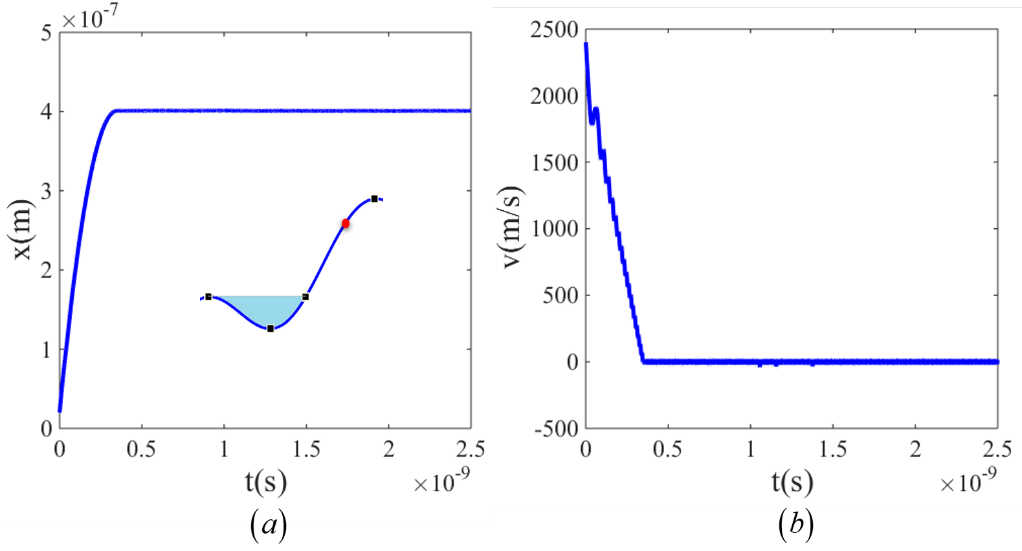


**Fig.A8** An example of the unlocking of particles when *v*_0_≠*v_wave_*. (a) The initial position of particle $P$ on the relative potential surface and the trajectory; (b) the unlocking velocity of particle $P$ with initial velocity $v_{0}=2400m/s$

From **Figs.A5** to **A8**, theoretical results of locking and unlocking cases all satisfy the conditions proposed in the paper.
